# Exploring techniques to assess stress-related physiological responses in captive African penguins^☆^

**DOI:** 10.1016/j.mex.2026.103860

**Published:** 2026-03-12

**Authors:** C. Currin, A. Ganswindt, L. Pichegru

**Affiliations:** a Department of Zoology, Nelson Mandela University, Gqeberha, South Africa; b Mammal Research Institute, University of Pretoria, South Africa; c Institute for Coastal and Marine Research, Nelson Mandela University, Gqeberha, South Africa

**Keywords:** Heart rate, Feather corticosterone, Plasma glucocorticoids, Urofaecal glucocorticoid metabolites

## Abstract

As animals experience a range of stressors, monitoring their stress response is important in conservation management and animal welfare practices. This study investigated possible techniques for evaluating stress-response in captive African penguins (*Spheniscus demersus*), including recording heart rate (HR) with a novel recorder inside a dummy egg, quantifying glucocorticoids in serum (sGC) and feathers (fGC) and glucocorticoid metabolites (ufGCM) in urofaeces.

● The HR recorders allowed for an assessment of immediate responses to stressors, however improvements in the design are necessary to record more consistent HR data.

● SGCs provide an assessment of an immediate stress response to acute stressors, however, due to the invasive nature of sampling this technique should only be used when blood is being drawn for medical reasons. UfGCM concentrations are less sensitive to short-term perturbations due to the accumulation of GC metabolites over longer periods, and due to the minimally invasive sampling procedure, allow for resampling, providing a useful tool for assessing long-term stress-related physiological response and welfare. FGC concentrations represent an integration of hypothalamic pituitary-adrenal activity over a period of days/weeks, however, whether this reflects circulating baseline levels or significant stressful events during feather growth is unclear and further research is needed to determine this.

## Graphical abstract

**Figure:**
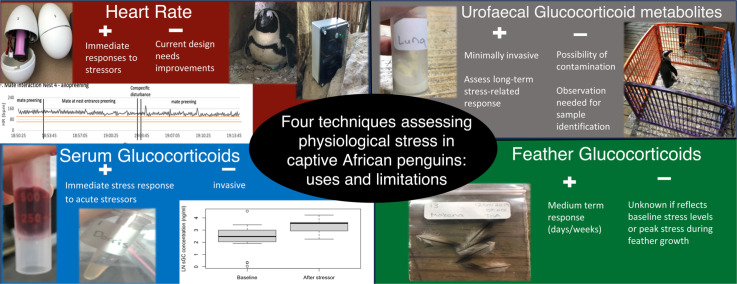

**Specifications table**

**Table:** 

**Subject area**	Agricultural and Biological Sciences
**More specific subject area**	Zoology
**Name of your method**	Assessing physiological stress responses using heart rate recordings, glucocorticoid and glucocorticoid metabolite concentrations
**Name and reference of original method**	Partly in: Mafunda PS, Maree L, Ganswindt A, Kotze A, van der Horst G (2020) Seasonal reproductive anatomy of the African penguin (*Spheniscus demersus*) with notes on endocrine correlates. IN: Animal Reproduction Science, 1(224):106,664; DOI:10.1016/j.anireprosci.2020.106664
**Resource availability**	All raw data are available on request.

## Background

All vertebrates respond to perceived stressors with a response regulated by the sympathetic-adrenal-medulla (SAM) axis and the hypothalamic pituitary-adrenal (HPA) axis [1]. A variety of stress-related parameters can be measured when evaluating how organisms respond to such challenges [2,3]. These parameters include the activity of the SAM axis (e.g., heart rate; [4]), and the HPA axis (glucocorticoids or its metabolites) [3,5]. Heart rate increases with activation of the SAM axis in response to stressors [1,6], but also as a result of orthostasis, exercise, changes in environmental temperature, eating a meal, or performing attention-requiring tasks [7]. Several methods exist for measuring heart rate response, including surgical implantation of a cardiac data logger [8], or attaching a cardiac transmitter on the back of animals [9], both methods being invasive [10]. Artificial eggs with hidden heart rate recording devices inside [11,12] are less invasive but limited to incubating birds.

Glucocorticoid (GC) production increases during activation of the HPA axis, thus glucocorticoids can quantify a physiological stress response [13,14]. GCs quantified using blood plasma or serum reveal an immediate “snapshot” of circulating GC concentrations [15] that also depends on the endogenous cycles, experiences immediately before sampling, and longer-term experience [15]. In addition, the secretion of GCs into the blood occurs in a pulsative fashion, therefore blood GC concentrations can change by a factor of 10 or more within minutes or seconds [16,17]. Less invasive methods utilize faeces, urine, or urofaeces (bird droppings) as a hormone matrix [[18], [19], [20]]. Faecal/urine GC metabolite concentrations represent an accumulation of GC metabolites over time (usually hours) [21]. Circulating GCs are processed by the liver and kidneys and excreted in metabolite form in the urine or faeces after a species-specific time delay, corresponding to the intestinal transit time [5,2]. The relatively non-invasive nature of faecal or urine sampling allows for re-sampling of the same individual without affecting the animals’ behaviour or endocrine status [18,22].

Recently, GC concentrations have been successfully extracted in keratinised tissue like feathers and hair [23,24]. GCs are deposited in the vascularised part of the feather during the growth phase, several days to weeks in most bird species, and represent an integration of HPA activity over this period [23] Studies suggested that feather GC concentrations may represent a measure of significant stress responses during feather growth, or a down-regulated concentration of GC [25,26]. Feather collection is minimally invasive, with short to no handling time, and as the calamus is removed prior to hormone extraction, feathers do not need to be plucked but can be clipped [23,24]. Feather GC concentrations remain stable after feather growth is completed, thus moulted feathers can be used, further reducing sampling disturbance. Samples can be stored at room temperature [27], where blood or faeces samples need to be frozen or extracted immediately.

This study has applied the above-mentioned methods for assessing physiological stress response in captive African penguins *Spheniscus demersus* to highlight their potential uses and limitations in this species.

## Method details

### Heart rate recording

#### Sample collection

This study took place in August 2021 at two captive facilities housing African penguins in South Africa, the Two Oceans Aquarium (TOA) in Cape Town, and the South African Foundation for the Conservation of Coastal Birds (SANCCOB) rehabilitation centre, in Gqeberha, where only the resident penguins in the home pen enclosure were used. Both facilities are open to the public with TOA experiencing much larger volumes of tourists than SANCCOB. At both facilities enclosures are entered by staff for cleaning and feeding throughout the day but staff rarely physically handle birds, only doing so for health checks annually or when individuals need treatment. Both facilities have breeding pairs which nest in artificial nest boxes. Neither facility allows eggs to come to term due to space constraints and a restriction on releasing captive bred chicks into the wild, therefore, all eggs laid are pricked and/or replaced with an artificial egg. A total of five nests were used in this study, two at SANCCOB and three at TOA, as very few penguins were incubating eggs at the time of the study.

#### Experimental equipment

**Fig. 1:**
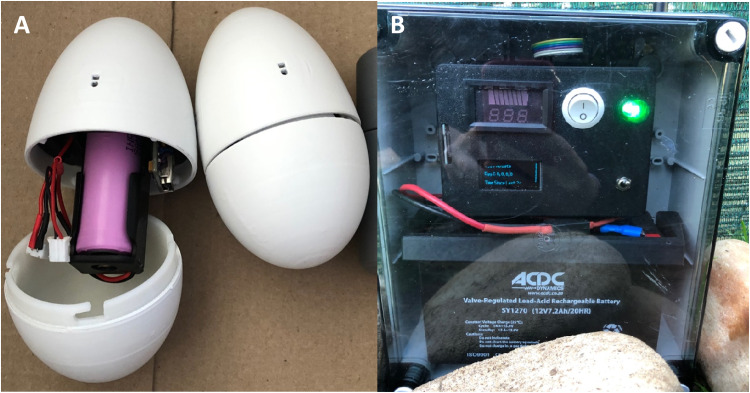
(A) A heart rate recorder hidden inside a dummy egg used to record heart rate response of incubating African penguins. (B) The base station used to receive the heart rate data and store it on a micro-SD card.

To monitor the heart rate of incubating penguins, a heart rate recorder hidden in a dummy egg was added to the clutch. The dummy egg consisted of an ATmega328P based microcontroller, 4 infrared pulse sensors [28] embedded in the outer casing, a 2.4 GHz wireless transceiver, an 18,650 lithium-ion battery and a voltage regulator circuit in a custom 3D printed enclosure designed to mimic an African penguin egg (88 mm x 55 mm in size) (Fig. 1A). The base station consisted of a microcontroller, a 2.4 GHz wireless transceiver, a micro-SD card reader, an OLED display, a lead-acid battery, and a voltage regulator circuit in a waterproof enclosure (Fig. 1B). The dummy egg heart rate recorder constantly monitored each of the four pulse sensors for the presence of a heartbeat and once a valid heartbeat was detected, the microcontroller sampled the heartbeat signal to calculate a current heart rate (Appendix A). This calculated heart rate was then transmitted every 5 s to the base station via the 2.4 GHz wireless transceiver module, along with an identifier for the dummy egg. This data was received by the base station and logged to the micro-SD card. An OLED screen on the base station displays the most recent data received by the base station, along with a time stamp. The power consumption of the dummy egg heart rate recorder was highly optimized so that the small lithium-ion battery with a capacity of 11.1 Wh could power the recorder for 5 continuous days of testing on a single charge. The system was validated and tested prior to deployment by comparing the resulting beats per minute (bpm) from the dummy egg to the simultaneous readings of a commercially available clip-on pulse oximeter in order to determine the accuracy of the dummy egg’s measurements. The dummy egg was originally designed by [29] and improvements, final assembly and creation of the base station were done by MandelaUni Autonomous Operations. A more detailed description of the components and operations is provided in Appendix A.

#### Experimental design

For each of the five focal nests, the dummy egg was switched on and placed in the chosen nest by lifting the back of the nest and rolling the dummy egg towards, or inserting it under, the penguin in the nest without removing the single artificial egg that was already in the nest. Dummy eggs were immediately incubated by all penguins. Once the penguin was incubating the dummy egg the receiver was switched on and placed in a location where it received strong, uninterrupted signal and was not disturbing any other penguins (preferably hidden from site), and the time was noted. To record different types of disturbances/stimuli occurring in the enclosure, a camera trap (Spypoint, Solar-Dark) was placed between three and seven meters from the focal nest depending on where an anchorage point was available with a direct line of sight. The camera was set to begin recording video immediately with no delay between recordings of 30 s videos. The memory card (32 GB) was replaced after approximately five hours and then left overnight to ensure enough space was available for all recordings. The experimental setup remained for a minimum of 48 h for each nest (Table 3 shows the time period over which HR was recorded for each nest), with battery life of the camera and receiver being the limiting factors. At the end of each trial the receiver and camera were switched off and the time noted, and the dummy egg was gently removed from under the incubating adult. No nest abandonment occurred during the monitoring period.

#### Heart rate telemetry

Heart rate (HR) was recorded in beats per minute (bpm) from each of the four sensors at five second intervals. A large amount of “noise” was present due to the movement of the incubating individual, which resulted in more than one sensor activating at a time; this noise needed to be distinguished from actual heart rate readings. Readings were considered as true heart rates when they were consistent from only one sensor for at least 30 s. Resting heart rate (RHR) was calculated as the average HR experienced during a period of undisturbed incubation of at least 30 s with no apparent stressors seen in the videos for the previous 10 min. Events (disturbances, natural stimuli/activities; Table 1) were identified from the videos and time of occurrence matched with the corresponding HR records. To determine levels of excitation reached in response to the different stimuli, the relative maximal increase in HR ( %RHR) was calculated as 100* (Max HR – RHR)/RHR for each HR response to a stimulus [30]. The duration of the HR response was not calculated as consistent HR readings were often only detected once a stimulus event had already begun or HR readings were interrupted by “noise” or loss of signal. As individual variation in RHR and HR responses was expected, but individual identity between partners within nests could not be determined, RHR and HR responses to similar types of stimuli were analyzed per nesting pair. Following [31], a tolerance band was calculated as two standard deviations from the mean RHR for each nesting pair, and when HR left this tolerance band for >15 s it was considered as excitation. Table B1 in Appendix B contains information for all HR response events recorded during the trials.

**Table 1:** Definitions of the disturbance events and natural stimuli that occur regularly within the environment of African penguins at two captive colonies (two oceans aquarium and SANCCOB EC).

Disturbances/stimuli	Description
Visitor presence (V)	Visitor presence in the area surrounding the enclosure, including talking loudly, screaming children, banging on the enclosure walls, that was detectable on the video footage.
Staff activities (S)	Activities of staff within the enclosure including cleaning, feeding, and moving around within the enclosure.
Self-maintenance behaviours (MB)	Behaviour an individual performs with no interaction with another individual, including preening and nest maintenance.
Conspecific disturbance (C)	Conspecifics (other than mate) moving or standing directly in front of the focal nest or fighting with other conspecifics in close proximity to the focal nest.
Mate interaction (MI)	Mate returning to nest, bringing nesting material, vocalizing with mate, or allopreening.

## Quantification of glucocorticoids and glucocorticoid metabolites

### Seruhm glucocorticoid concentrations

#### Sample collection

A total of 35 individuals were used for sample collection from both TOA and SANCCOB, and initial baseline serum, urofaecal and feather samples were collected sequentially from individuals where possible. The identity, age, and sex of each individual sampled were noted. A total of 28 individual blood samples were collected from TOA (*N* = 16, June to September 2019) and SANCCOB (*N* = 12, September 2019). These samples were considered to represent baseline concentrations of sGC as they were collected prior to any intentional stressors and within 3–5 min of capture [32]. All blood samples were collected between 7h00 - 12h00. Individuals were captured following standard protocols (i.e., grabbed behind the neck and gently lifted from the nest while holding the legs) and restrained between the seated handler’s legs at mid-flipper height, with the feet exposed for blood collection and the head restrained. The birds were restrained for no longer than 10 min in total while all samples (blood, feathers and urofeaces) were collected sequentially, i.e. blood samples were collected within < 5 min of capture. Approximately 0.5 ml of blood was collected using venipuncture on the foot vein with 21 G x 5/8 hypodermic needles. Blood was left to clot for 60 min and then centrifuged at 1300x g for 15 min to separate the serum, which was subsequently decanted into an Eppendorf tube and frozen at -20 °C. A further 15 blood samples were collected in March 2020 from 15 birds at TOA during a presumed stressful health assessment event, where all individuals were captured and placed in a pen together and taken one by one from the pen and bled (individual time in the pen varied from 13 – 276 min; Table 3). The time from removing the bird from the pen till the needle was drawn after blood collection ranged from 3–5 min. The 15 birds were individually identified to allow a comparison of respective GC values from the baseline samples obtained in 2019 and samples obtained after the health assessment in March 2020 (Table 3). Frozen serum samples were sent to the Endocrine Research Laboratory of the University of Pretoria for further processing.

#### GC quantification

Serum GC concentrations were determined using an enzyme immunoassay for corticosterone, as described by [33]. Assay specificities including cross-reactivities of the antibody are given in [33]. The sensitivity of the assay was 80 ng/ml and the intra-assay coefficients of variation (CV) of high and low concentration controls was 4.57 % and 5.74 %. The inter-assay CV of high and low concentration controls was 5.02 % and 9.24 %. Serial dilutions of serum samples gave displacement curves that were parallel to the respective standard curve with a relative variation of the slope of the trend lines of *<* 8 %.

### Urofaecal glucocorticoid metabolite concentrations

#### Sample collection

A total of 22 individual urofaecal samples were collected to assess baseline GC metabolite concentrations, 19 at TOA (June to September 2019) and three at SANCCOB (September 2019). Penguins were restrained over a clean plastic sheet in order to collect any excreted urofaecal samples. A syringe was used to draw up only the faecal part of the guano (between 1–5 ml), which was then placed in a 5 ml plastic tube and stored at -20 °C until further processing. To investigate ufGCM fluctuations over time, including a potential increase in ufGCM concentration after exposure to a presumed stressful event of capture, additional urofaecal samples were repeatedly collected over seven hours (8h00 – 15h00) from seven individuals (four males and three females) at TOA (*n* = 4 per individual) during August and September 2019. For that the penguins were captured and placed in individual pens on plastic sheeting for a maximum of five minutes, representing a stressful event. After collecting an initial sample (*t* = 0), the penguin was returned to the enclosure, and sample collection continued during set time intervals (2–3; 4–5; and 6–7 h) in the enclosure by an observer with minimal disturbance to the penguin. Frozen samples were sent to the Endocrine Research Laboratory of the University of Pretoria for further processing.

#### Steroid extraction and ufGCM quantification

Frozen urofaecal samples were lyophilised, pulverised, and sieved through a wire-mesh strainer to remove fibrous material. Between 0.050 – 0.055 g of faecal powder from each sample was then mixed with 1.5 ml of 80 % ethanol, vortexed for 15 min and centrifuged for 10 min at 1500 x g for steroid extraction. The supernatant was decadent into microtubes and stored at -20 °C until analysis [34]. Steroid extracts were analysed for ufGCM concentrations using a tetrahydrocorticosterone enzyme immunoassay (EIA), previously established for quantifying ufGC metabolites in African penguin droppings [34]. Assay specificities including cross-reactivities of the antibody are provided in [35]. The sensitivity of the assay was 9.6 ng/g dry faecal mass (DW) and the intra-assay CV of high and low concentration controls was 3.36 % and 4.38 %. The inter-assay CV of high and low concentration controls was 7.36 % and 8.76 %. Serial dilutions of extracted samples gave displacement curves that were parallel to the respective standard curve (relative variation of the slope of the trend lines *<* 5 %).

**Fig. 3:**
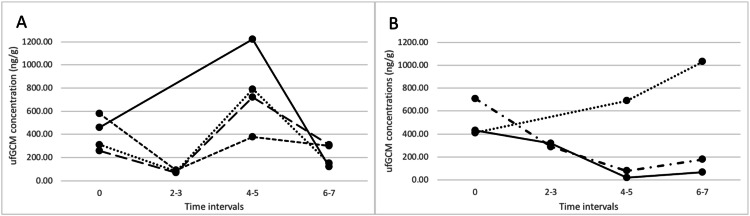
Temporal patterns of urofaecal glucocorticoid metabolite (ufGCM) concentrations measured in male, *n* = 4 (A), and female, *n* = 3 (B) captive African penguins at Two Oceans Aquarium in Cape Town, beginning with a baseline measure before a stressor (capture) was experienced and measured at regular time intervals after the stressor (2–3 h, 4–5 h, and 6–7 h).

When analysing the additional repeated samples collected from the seven individuals at TOA, temporal changes in penguins’ ufGCM concentrations were apparent, revealing individual variation in overall concentrations, but also sex-related patterns (Fig. 3). In males, concentrations initially decreased, and then peaked after 4–5 h, which may reflect the response to capture, and then decreased again (Fig. 3A). In two of the females ufGCM generally decreased through time then increased slightly after 6–7 h, while the third female’s ufGCM steadily increased up until the 6–7 h interval, potentially reflecting a response to capture sooner than the other females (Fig. 3B). These results highlight the importance of considering the time of day and sex when analysing ufGCM concentrations in captive African penguins.

### Feather glucocorticoids

#### Sample collection

A total of 35 individual feather samples were collected to assess baseline GC concentrations, 23 at TOA (June to September 2019) and 12 at SANCCOB (September 2019). Two of the 12 individuals at SANCCOB had been moved immediately prior to their moult to an unfamiliar enclosure where they remained until after their moult, subjecting them to a presumed stressful event during the feather growth period prior to sample collection for this study, providing two likely compromised individuals as a comparison to the uncompromised individuals (this was not done as a component of this study but as a result of renovations at SANCCOB). Five feathers were collected from each individual while they were restrained for blood collection, by pulling single feathers upright and cutting them directly above the skin with a pair of surgical scissors, and were taken from five separate places on the back of the bird to avoid compromising the birds’ waterproofing. Feathers were stored in a pre-labelled plastic bag at room temperature and sent to the Endocrine Research Laboratory of the University of Pretoria for further processing.

#### Steroid extraction and hormone analyses

Feather samples were liquidised prior to analysis following [36]. After completion of the liquifying process, the samples were centrifuged for 10 min at 1500 x g to separate the remaining solid material. The supernatant was pipetted just below the surface, transferred to a labelled micro centrifuge tube and stored at -20 °C until hormone analysis. Steroid extracts were analysed for immunoreactive corticosterone concentrations using a corticosterone EIA following previously established protocols by [37]. Assay sensitivity was 28 ng/g feather mass. Parallelism was not determined for the feather extracts because only one dilution was used for the hormone analysis for all samples.

## Method validation

### Performance of the heart rate monitor egg

Of the 236.75 h over which HR was recorded, the dummy egg heart rate recorder was able to detect reliable HR readings only 5 % of the time (Table 2). The reliability of the dummy egg to record heart rate varied between the nesting pairs (Table 2). For majority of the time there was no HR detected from the dummy egg at all, and for the remaining time there was “noise” from the incubating individual moving.

**Table 2:** Table showing the performance values for the heart rate recorder dummy egg used to record HR response in captive African penguins at two oceans aquarium and SANCCOB.

Nest	Total time of trial (s)	Total time of HR readings recorded with corresponding video (s)	Total time of useable HR readings recorded (s)	Percent of total recorded time with useable HR readings ( %)
1	86,400	1430	1575	2
2	192,300	1610	3545	2
3	228,000	2995	5315	2
4	172,800	7760	12,825	7
5	172,800	10,305	20,440	12
Total	**852,300**	**24,100**	**43,700**	**5**

**Fig. 2:**
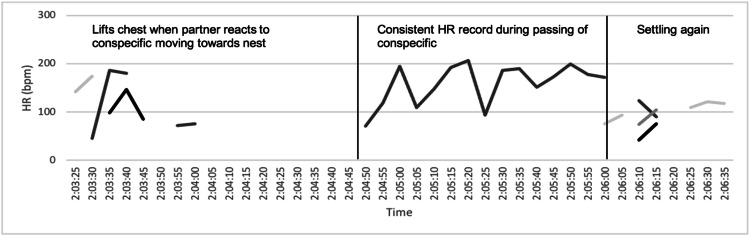
Heart rate recording using a heart rate recorder in a dummy egg to record heart rates in captive incubating African penguins showing “noise”, i.e., interrupted signal or erratic signal, and a segment of detectable heart rate while a stimulus was present. Differently shaded lines represent each of the four infrared sensors embedded in the dummy eggs exterior.

“Noise” occurred when more than one sensor was activated simultaneously due to the movement of the incubating individual. In this example (Fig. 2), a conspecific ran towards and passed the focal nest resulting in the incubating individual raising their chest as the conspecific approached, intermittently interrupting the signal from the dummy egg. Consistent HR readings were recorded for a brief time (75 s) as the conspecific passed the nest and then more “noise” was detected as the incubating individual settled back onto the dummy egg. This was a common occurrence as individuals often reacted behaviourally in response to disturbances, particularly when the disturbance was close to their nest, generally resulting in a disruption of the signal from the dummy egg. Both “noise” or loss of signal made measuring changes in HR for the duration of an entire response difficult as an initial heart rate immediately prior to the disturbance was not always detected and signal was often lost before HR returned to RHR levels. Examples of other behaviours which resulted in “noise” or a loss of signal include preening, nest maintenance, shifting position, rotating the eggs, aggressively reaching forwards, or moving when mates entered the nest.

### Serum glucocorticoid concentrations

The assay was biologically validated for sGC concentrations by comparing individual baseline sGC concentrations to the respective sGC concentrations after a presumed stressful event (capture and restraint for health assessment) using a paired *t*-test, revealing a significant (*n* = 15, *t* = -3.338, *p* = 0.005) overall increase in sGC levels of 68 % (baseline: 19.12 ± 22.49 ng/ml (mean ± SD) vs after capture and restraint: 32.15 ± 16.12 ng/ml).

### Feather glucocorticoids

To evaluate if the chosen EIA detected biologically meaningful alterations in fGC concentrations, the respective fGC concentrations in two compromised individuals (subjected to a stressful situation of relocation to unfamiliar surroundings immediately prior to and during their moult) were compared to the overall population baseline fGC concentrations using a student’s *t*-test and were found to be on average 31 % higher (1.64 ± 0.24 ug/g vs 2.36 ± 0.3 ug/g; *t* = -4.106, df = 33, *p* = 0.0002).

## Limitations

These techniques all have potential for evaluating stress and monitoring welfare in African penguin populations, however, which technique is best applied will depend on the specific circumstances and limitations outlined inTable 4.

**Table 3:** Comparison of captive African penguin baseline serum glucocorticoid concentrations (sGC) with concentrations after a presumed stressful health assessment event, where individuals were captured and placed in a pen together and taken one by one from the pen and bled (individual time in the pen provided in the first column).

Time in pen (min)	Penguin ID	Baseline sGC conc. (µg/ml)	Stressed sGC conc. (µg/ml)
13	Labamba	16.05	35.39
27	Laduma	1.4	51.54
29	Zuki	26.61	36.52
34	Tasmyn	23.28	50.13
39	Agape	94.41	68.51
53	Neptune	6.74	37.65
50	Ayoba	8.89	34.83
59	Quinn	30.79	17.00
59	Dorris	1.38	20.68
76	Diesel	1.04	10.54
91	Chuck	8.93	37.27
122	Flippy	11.79	9.58
151	Gaia	15.47	17.56
161	Faraday	17.17	27.51
276	Anuli	8.63	19.81

**Table 4:** Comparison of four possible techniques for evaluating physiological stress response in African penguins.

	Techniques for evaluating physiological stress response			
	Serum Glucocorticoids	Urofaecal Glucocorticoid Metabolites	Feather Glucocorticoids	Heart rate using a heart rate recorder in a dummy egg
**Invasiveness**	Highly invasive.	Minimally invasive.	Minimally invasive if collecting moulted feathers. Fairly invasive if capturing and clipping feathers or plucking feathers.	Minimally invasive, only requiring small disturbance when placing and removing dummy egg.
**Timeframe**	Immediate.	Typically, hours, depending on intestinal transit time.	Days or months, depending on feather growth rate.	Immediate.
**Practicality of sample collection or storage, and implementation of technique**	Potential for harm to both handler and organism during sampling process. The process of capturing individuals can result in disturbance of other individuals, especially in wild populations. Samples need to be kept frozen to prevent degradation of glucocorticoids.	Easy to obtain samples but possible difficulty in identifying which individual the sample belongs to when using wild individuals. Samples need to be kept frozen.	Easy to obtain samples when moulted feathers are used. Potential harm to both handler and organism if feathers are clipped or plucked.	Easy to place dummy egg. Potential for the dummy egg to be rejected, although this is minimal.
**Opportunity for repeated sampling (longitudinal or cross-sectional)**	Due to the invasive nature of the sampling procedure, repeated sample collection from the same individual over time would likely result in unnecessary stress and potentially skew subsequent GC concentrations, unless evaluating the response to repeated handling was the objective	Able to collect repeated samples from the same individual over time but requires constant observation during sample collections to ascertain that the sample belongs to the same individual. Possible to collect multiple samples from several individuals easily.	As GCs are only depositing during the growth phase of the feather, in organisms with catastrophic moults collecting feathers is limited to one collection per individual per moulting period (usually a year). In organisms that moult continuously feathers can be collected multiple times from the same individual but is challenging in wild organisms as constant observation would be needed to identify the owner of the feathers.	Repeated recording of the same individual is possible but is limited to within the incubating period of the breeding season.
**Reliability of results**	Easily influenced by the sampling procedure, as well as possible unknown factors prior to sampling.	Not influenced by sample collection but may be influenced by unknown factors prior to sampling.	Represents an accumulation of glucocorticoids over feather growth period, thus is not influenced by sampling process. Uncertainty as to whether feather glucocorticoids represent baseline levels, large stress events, or down-regulated levels during feather growth.	Possibility for large amounts of disturbance in heart rate readings due to movement of individual while incubating. Heart rate can also fluctuate as a result of orthostasis, exercise, changes in environmental temperature, eating a meal, or performing attention-requiring tasks, making it potentially difficult to link heart rate variations to specific variables at times.
**Limiting factors**	Amount of blood available may be limiting (e.g., small species).	Potential for contamination from substrate if collected from wild individuals.	If using moulted feathers, sample collection is limited to the moulting period. Short-term stressors cannot be assessed using moulted feathers.	Can only be used for incubating individuals and is limited to the breeding season. Loss of signal due to loss of contact between individual and sensors.

While the dummy egg heart rate recorder had limitations in its current design, it was still able to record valuable HR data during different disturbances and stimuli. An improved design would need to increase the contact between the brood pouch and a single sensor at a time to detect HR, for example by weighting one side of the dummy egg so that one sensor will always point upward [12,38]. In addition, while an HR recorder in an egg limits its application to the incubating period, it remains very useful as this is a time when individuals are less likely to show an overt behavioural response to disturbances. Despite the limitations of the dummy egg HR recorders their use will be preferred in most situations over other methods of obtaining HR measurements, such as implanted cardiac data loggers or cardiac transmitters fixed to individuals [8,9], as it is significantly less invasive, only requiring disturbance during placement of the dummy egg. Once improved it could be used to measure HR responses to stressors in both captive and wild colonies, allowing identification of what disturbances may act as both short-term and long-term stressors in African penguins.

Measuring GC concentrations from blood serum/plasma allows for an evaluation of an immediate response to a stressor, which is not possible to obtain using urofaeces or feathers but is valuable when assessing the effect of acute stressors in an organism’s environment. If using this technique, the limitations need to be considered, which includes; the total amount of blood available [18], difficulty in obtaining the samples, possible risk to the animal and/or sampling person [2], and circulating GC levels can change rapidly in response to stress and thus, the sampling process generally results in increased circulating GC levels, making an unbiased sample difficult to obtain [39,2,19], and the act of repeated blood collection would cause greater levels of stress, which would be counter-productive in improving welfare. Due to the invasive nature and difficulty of sample collection, this technique should preferably only be applied when blood is already being drawn for other purposes, such as vital health assessments. In these instances, a baseline sample could be obtained if collected within the 3–5-minute window before GC levels increase in response to handling, and another sample collected after 5 min while the individual is still restrained, should reflect any increase in GC in response to handling and phlebotomy. When deciding if this technique is necessary the value of the information gained should be weighed against the possible negative impacts of collecting a blood sample.

Due to the minimally invasive nature of sample collection when using urofaeces it is possible to collect repeated samples from the same individuals over time, as well as multiple samples from different individuals within a colony without causing much disturbance. This provides a valuable tool for monitoring stress levels in both captive and wild colonies over long term periods, which could help guide management decisions with regards to limiting potential stressors. As shown in this study it may also be possible to link specific stressful events with a rise in ufGCM concentrations under controlled conditions, (i.e., when the exact time of the stressful event is known), and this could provide a means to assess the magnitude of response to particular stressors without having to draw blood.

Using keratinised tissue (feathers) to measure GC concentrations is still in its infancy and there are still many uncertainties as to how GC is deposited in feathers, as well as several limitations, which includes the inability to assess short-term stressors, and the restriction of measurement of GC levels to the time in which feathers grow [[39], [40]]. If collecting moulted feathers from catastrophic moulters (such as penguins), sample collection is restricted to the time at which moulting occurs, which is usually once a year. Collecting moulted feathers from birds that moult continuously can be challenging in wild populations as it is not always possible to identify the owner of the feathers unless under constant observation. However, the potential for using feather GC as a means to evaluate physiological stress responses and welfare in African penguin populations is promising. Feather GC concentration should be less sensitive to short term perturbations, due to the longer timeframe over which GCs are deposited into feathers, representing an integration of HPA activity over a period of days or weeks. This study showed that a large stressful event was evident in the fGC concentrations of two individuals indicating that it is possible to detect the presence of stressors in an environment using fGC concentrations. Before feather GC can be used to make inferences about stress responses in African penguins more research needs to be done to determine whether feather GC reflects circulating baseline levels or significant stressful events during the feather growth period.

## Ethics statements

Ethical clearance was obtained for research analysed in this study from Nelson Mandela University’s Research Ethics Committee (REC-A), which complies with or the National Institutes of Health guide for the care and use of laboratory animals (NIH Publications No 8023, revised 1978), for the period of 2019–2021. The ethics clearance reference number is **A18-SCI-ZOO-008**.

## CRediT authorship contribution statement

**C. Currin:** Conceptualization, Methodology, Formal analysis, Investigation, Writing – original draft, Visualization. **A. Ganswindt:** Methodology, Resources, Writing – review & editing, Supervision. **L. Pichegru:** Conceptualization, Methodology, Resources, Writing – review & editing, Supervision, Funding acquisition.

## Declaration of competing interest

The authors declare that they have no known competing financial interests or personal relationships that could have appeared to influence the work reported in this paper.

## Figures and Tables

**Figure:**
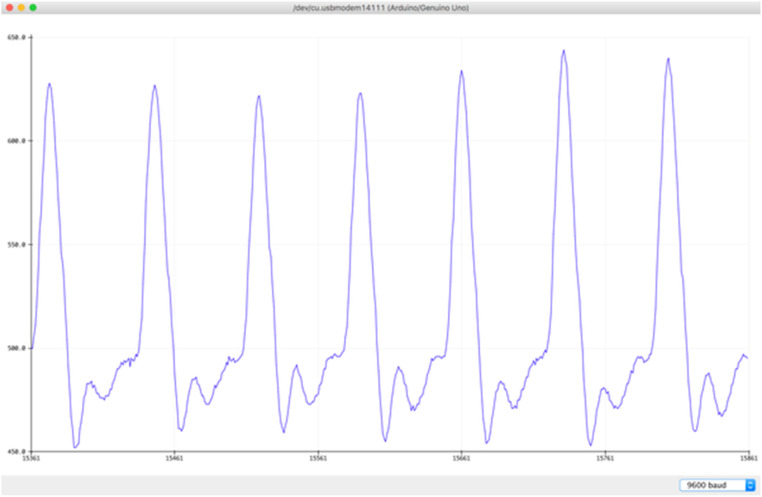
▒.

**Table B1:** Table showing all the HR responses recorded for five nesting pairs of African penguins exposed to various human disturbances and natural stimuli present in a captive environment, using a heart rate recorder disguised in a dummy egg.

Nest	Type of disturbance/stimulus	Avg HR (bpm)	HR MAX (bpm)	%RHR	Recorded time period of response (s)	Time spent above tolerance band (s)
1	RHR	64.09	76		355	0
	RHR	70.38	97		470	0
	RHR	64.69	82		155	0
	RHR	85.36	108		55	5
	RHR	87	99		195	0
	Staff activity (feeding)	141.43	185	116.48	35	25
	Conspecific disturbance (standing outside nest)	155.27	218	155.09	80	70
	Conspecific disturbance (standing outside nest)	186.31	225	163.28	85	75
2	RHR	90.03	107		320	10
	RHR	88.16	96		215	0
	Staff activity (feeding)	99.5	225	112.97	40	10
	Staff activity (in enclosure)	155.86	182	163.28	35	35
	Self-maintenance behaviour (nest maintenance)	149.43	207	142.22	35	30
	Self-maintenance behaviour (preening)	126.86	166	94.24	35	25
	Self-maintenance behaviour (preening)	111.5	192	124.67	40	20
	Conspecific disturbance (standing outside nest)	122.38	217	153.92	45	25
	Conspecific disturbance (standing outside focal nest)	137.12	217	153.92	165	125
	Conspecific disturbance (standing outside nest)	116	191	123.5	40	25
	Conspecific disturbance (standing outside focal nest)	122.83	207	142.22	30	15
	Mate interaction (mate returns to nest)	133.5	232	171.47	35	20
	Mate interaction (allopreening)	121.29	174	103.6	45	25
	Mate interaction (partner returns to nest)	143.74	222	159.77	215	100
	Mate interaction (mate returns to nest)	145.21	213	149.24	70	50
	Mate interaction (vocalising in nest)	132.56	232	171.47	85	50
	Mate interaction (allopreening)	114.11	218	155.09	160	50
3	RHR	75.93	106		285	5
	Visitor presence (loud talking)	81.57	145	69.67	2010	90
	Staff activity (feeding)	158.67	229	167.96	90	75
	Conspecific disturbance (vocalising)	185.63	236	176.15	30	25
	Conspecific disturbance (standing outside focal nest)	182.24	234	173.81	295	245
	Conspecific disturbance (fighting outside focal nest)	186.64	234	173.81	100	80
	Conspecific disturbance (standing outside focal nest)	175	218	155.09	90	75
	Mate interaction (allopreening)	173	202	136.37	65	60
	Mate interaction (mate returns to nest)	138.33	217	153.92	30	20
4	RHR	89.89	107		455	10
	RHR	85.72	99		90	0
	Visitor presence (kid shouting loudly)	100.5	169	97.75	30	10
	Visitor presence (loud talking)	110.75	182	112.97	40	15
	Visitor presence (loud talking)	127.7	155	81.37	315	275
	Visitor presence (loud talking)	97.95	123	43.93	110	60
	Visitor presence (loud talking)	130.98	177	107.11	5000	4980
	Staff activity (cleaning enclosure)	107.88	159	86.05	50	20
	Staff activity (standing close to nest)	101.79	129	50.95	155	65
	Conspecific disturbance (standing outside focal nest)	154.67	220	157.43	30	25
	Conspecific disturbance (standing outside focal nest)	109.78	161	88.39	45	20
	Mate interaction (allopreening)	126.7	182	112.97	1440	1410
5	RHR	81.81	103		80	0
	RHR	90.28	109		185	5
	RHR	86.88	97		85	0
	RHR	91.98	123		1380	75
	RHR	91.95	108		295	15
	RHR	92.36	110		350	10
	RHR	93.15	112		780	40
	RHR	97.07	119		215	30
	RHR	96.13	121		300	40
	RHR	88.03	104		305	0
	RHR	84.61	102		270	0
	RHR	84.53	97		160	0
	Visitor presence (loud talking)	104.89	128	49.78	225	105
	Visitor presence (loud talking)	121.92	179	109.45	180	170
	Visitor presence (loud talking)	99.52	141	64.99	500	125
	Visitor presence (loud talking)	85.56	131	53.29	630	35
	Staff activity (feeding)	98.1	126	47.44	210	65
	Staff activity (feeding)	98.02	121	41.59	230	60
	Staff activity (feeding)	113.54	170	98.92	1555	1035
	Staff activity (cleaning enclosure)	83.56	131	53.29	390	20
	Staff activity (cleaning enclosure)	101.67	128	49.78	315	185
	Self-maintenance behaviour (nest maintenance)	110.03	144	68.5	180	140
	Self-maintenance behaviour (nest maintenance)	91.13	132	54.46	30	10
	Self-maintenance behaviour (nest maintenance)	124.53	198	131.69	240	85
	Self-maintenance (preening)	109.15	144	68.5	305	235
	Conspecific disturbance (standing outside focal nest)	118.4	170	98.92	440	290
	Conspecific disturbance (standing outside focal nest)	158.5	206	141.05	75	65
	Mate interaction (mate returns to nest)	152.21	213	149.24	160	125
	Mate interaction (mate brings nesting material)	140.15	209	144.56	65	45
	Mate interaction (mate returns to nest)	128.03	155	81.37	170	170

## Data Availability

Data will be made available on request.

## Appendix A

The dummy egg made use of a Pulse Sensor, manufactured by World Famous Electronics (https://pulsesensor.com/) to record the heartbeat data. This product makes use of an ambient light sensor from Avago (APDS-9008), a green super bright reverse mount LED from Kingbright (AM2520ZGC09), and other complementary components in order to detect the change in reflected light when blood pumps through the capillary tissues (World Famous Electronics, 2018).

The output of the pulse sensor was sampled at 500 Hz, an example of this raw output signal can be seen below (https://pulsesensor.com/pages/code-and-guide).

The microcontroller then found the exact time of each peak of the pulse signal and calculated the Interbeat Interval (IBI), or time between each beat, in milliseconds. This IBI value was then converted to beats per minute (bpm) with the formula:

$$bpm=\frac{60}{IBI}$$

If more information is needed on the design or components of the dummy egg heart rate recorder or base station, J. Steele from MandelaUni Autonomous Operations can be contacted at s214048438@mandela.ac.za.

## Appendix B

Table B1
